# Continuously Frequency-Tuneable Plasmonic Structures for Terahertz Bio-sensing and Spectroscopy

**DOI:** 10.1038/s41598-019-39015-6

**Published:** 2019-03-05

**Authors:** Xiangying Deng, Leyang Li, Mitsuhiro Enomoto, Yukio Kawano

**Affiliations:** 1 Laboratory for Future Interdisciplinary Research of Science and Technology, Department of Electrical and Electronic Engineering, Tokyo Institute of Technology 2-12-1, Ookayama, Meguro-ku, Tokyo 152-8552 Japan; 2 Department of Orthopaedic Surgery, Tokyo Medical and Dental University, 1-5-45 Yushima, Bunkyo-ku, Tokyo 113-8519 Japan

## Abstract

Plasmon-based devices are powerful for use in highly sensitive evanescent-field detection and analysis, but they exhibit the problem of limited frequency tunability for fixed structures. This feature causes problems in the multi-frequency investigations required for materials characterization, bio-related research, etc. Here, we propose and fabricate a spiral-shaped plasmonic structure that enables a continuous frequency-tuneable evanescent-field concentration in the terahertz (THz) region with simple operation. The device also increases the electric field intensity at the subwavelength aperture, thus significantly amplifying the transmission. Highly tuneable transmission bands are observed by simply rotating the spiral plasmonic structure, which are in good agreement with the behaviour expected from electromagnetic simulation. Medical examinations are performed by measuring the interactions between the frequency-tuneable plasmons and bio-samples, which enables observing distinct tissue-dependent transmission spectra and images. The developed device simultaneously offers the advantages of both plasmonic devices and frequency-tuneable devices, which can increase the availability and versatility of evanescent-field THz sensing and analysis. The mechanism presented will shed light on THz plasmonics and motivate the implementation of a variety of applications based on plasmon-mediated THz technologies.

## Introduction

Due to their unique nature, terahertz (THz)-based technologies show strong potential for use as analytical tools for a variety of applications, including security, medicine, food quality inspection, electronics, and astro-observation^1–7^. One of the major obstacles in the exploration of THz applications is the longer wavelength of THz waves relative to infrared, visible, and ultraviolet light. The scale of the THz wavelength (300 μm at 1 THz) prevents detailed studies of tiny samples because the power received by such samples significantly decreases as the sample size is reduced below the subwavelength scale. This issue is particularly critical for examining micro/nanoscale objects such as nanomaterials, cells, and molecules, which hinder the capability and usefulness of THz measurements. This issue highlights the importance of highly efficient THz wave concentration and coupling with such samples.

The use of subwavelength apertures together with surface modification to generate high electromagnetic wave transmission and an extraordinary electric field concentration provides one possible solution to these problems^8–13^. These modifications are based on a coupling mechanism involving the generation and propagation of surface plasmon polaritons (SPPs) along a metal-dielectric interface^14^, a graphene waveguide^15^, and a nanoscale plasmonic resonator^16^, which enables the tight confinement of electromagnetic waves. In the THz region, spoof surface plasmons (SSPs), which are electromagnetic waves that mimic SPPs, can also take advantage of these subwavelength structures and increase the transmission through the aperture^17,18^. Though such “plasmonic apertures” display vastly higher transmission than plain apertures beyond the diffraction limit, a main shortcoming for such plasmonic-based devices is that once fabricated, the resonant frequency for such structures is fixed to a single frequency with poor frequency tunability^19^. This feature limits the useful operating range for THz plasmonic devices, despite the wide potential of plasmon-mediated applications.

In this paper, we present a continuously frequency-tuneable plasmonic-based THz structure. To realize such a tuneable plasmonic device, we propose and fabricate a spiral bull’s eye (SBE) structure with a subwavelength aperture surrounded by continuously varied, concentric plasmonic grooves. Because of the smoothly varied grooves, the groove period continuously changes with the diameter direction, resulting in continuously frequency-tuneable characteristics. To simultaneously enable THz field concentration and frequency tunability, a Siemens-star aperture was utilized instead of a conventional subwavelength circular aperture. We demonstrated that in the SBE structure, the frequency of the THz transmission peak can be tuned according to the angle of polarization (AoP) of the incident THz wave relative to the surface of the SBE structure. Thus, the transmitted frequency can be easily selected by simply changing the rotational direction of the SBE structure. We examined the transmission spectra of a medicine tablet using the SBE and verified its frequency-tuning ability and applicability to local THz spectroscopy. Evanescent-field bio-sensing was then performed to further confirm the advantages of the SBE for medical examinations, leading to spectroscopic and spatial identification of mouse tissues by observing the tissue-dependent THz signals. We expect that the developed spiral plasmonic structures can be applied to arbitrarily tuneable THz plasmonic devices and will promote applications that require multi-frequency investigations, such as medical examination, molecular research, and materials and device characterization.

**Figure 1:**
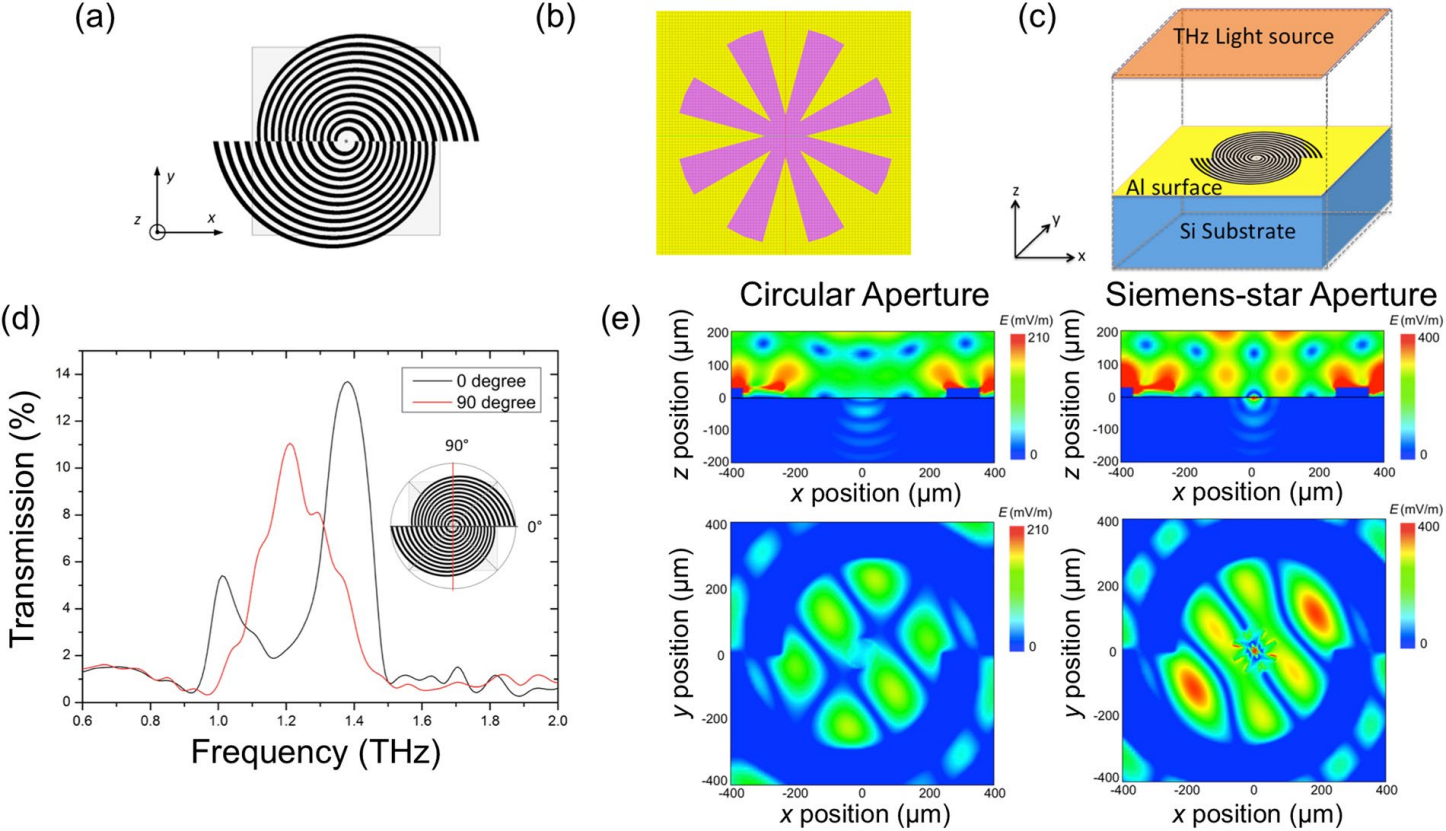
Device structure and simulation results. (**a**) Illustration of the SBE structure. (**b**) Simulation model for the eight-tip Siemens-star aperture. (**c**) Simulation model for the SBE structure. (**d**) Simulated transmission spectra for the Al-based SBE (**e**) FDTD simulations of the electric field component (*E*) distribution for the circular subwavelength aperture (left) and the eight-tip Siemens-star aperture (right). The colour bar values are given on a logarithmic scale and normalized by the THz source power.

We designed the SBE structure so that the frequency range lies between 1 THz and 1.4 THz (Fig. 1(a)). When the incident linearly polarized THz wave perpendicularly reaches the air-metal interface, SSP waves are excited within a designed spectral band, thus increasing the transmission intensity through the subwavelength aperture. To further improve the surface electric field concentration at the aperture of the SBE structure and induce multi-frequency coupling, a Siemens-star aperture (Fig. 1(b)) was used instead of the conventional circular subwavelength aperture. Special aperture shapes, such as a bow-tie aperture with sharp tips, have previously been shown to exhibit highly increased electric fields near the tips^20^. Despite the advantages of the bow-tie aperture, it is also highly dependent on the polarization, allowing only one directional SSP wave to propagate and transmit through the aperture. The Siemens-star aperture has more tips than the bow-tie aperture^21^ and thus enables more possible AoPs.

We numerically investigated the THz transmission spectra for the SBE structure using a finite-difference time-domain (FDTD) method with a non-uniform grid mesh. As depicted in the simulation model in Fig. 1(c), the SBE structure studied here has concentric grooves with continuously varying diameters. We can therefore expect that the transmission spectrum is determined by the AoP of the incident linearly polarized wave. The simulated transmission spectra in Fig. 1(d) reveal that the SBE exhibited enhanced transmission at approximately 1 THz and 1.4 THz at the AoP of 0° (*x*-polarization), whose direction corresponds to the interface of the two half-spiral sections. After rotating the AoP of the incident THz wave by 90° (y-polarization), the transmission peak shifted to approximately 1.2 THz, showing high frequency selectivity depending upon the AoP, as well as the possibility of tuning the transmitted frequency continuously in a specific spectral range.

In this work, the Siemens-star aperture was designed to have an outer diameter of 150 μm and 8 tips with a tip angle of 30°. Figure 1(e) displays FDTD simulations for the electric field component *E* distribution. The eight-tip Siemens-star aperture was shown to be approximately 3–5 times more efficient in generating high electric field enhancement than the circular subwavelength aperture. The reason for this field enhancement is that the sharp tips at the centre more strongly concentrate electric fields near the tips, compared to the circular aperture without such sharp structures. The highest *E* value for the Siemens-star aperture in this work is located near the centre of the structure. The electric field distribution within the Siemens-star aperture on the *z* = 0 and *y* = 0 planes is also different from that found for the circular subwavelength aperture; the former largely contributes to the electric field enhancement at both the exit interface of the central aperture and the area within the first groove circle.

**Figure 2:**
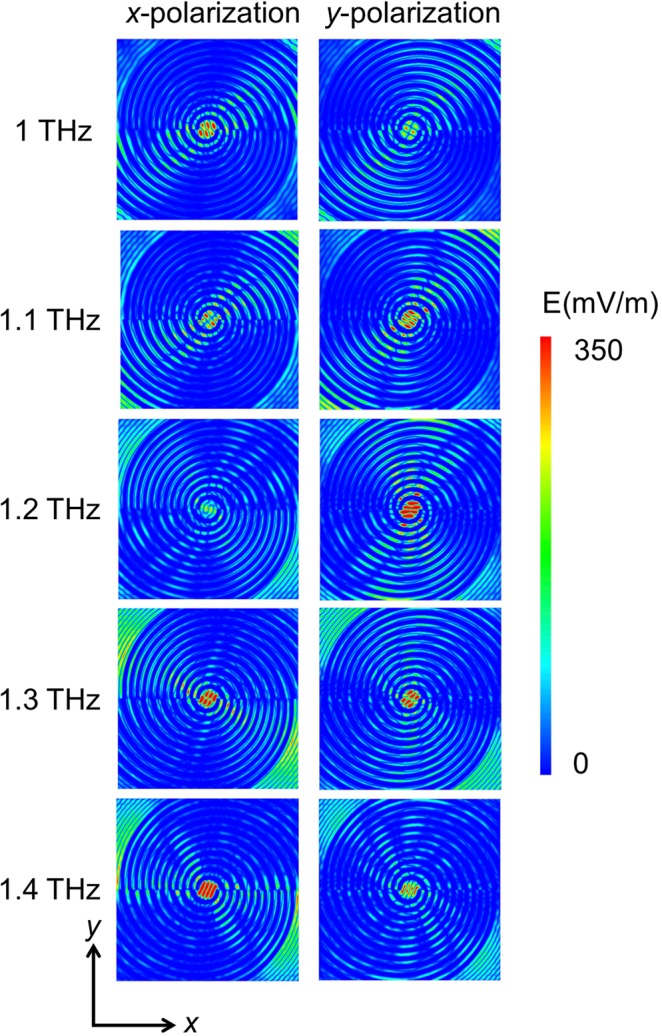
Simulated electric field distribution at different frequencies for *x*-polarization and *y*-polarization.

The simulated electric field distribution of the proposed SBE for *x*-polarization and *y*-polarization at different frequencies is mapped out in Fig. 2. It was seen that the resonance area shifted along the grooves as the frequency changed from 1 THz to 1.4 THz: The result for *x*-polarization exhibits higher field concentration at the central aperture at 1 THz and 1.4 THz, while the result for *y*-polarization exhibits higher field concentration at the central aperture at 1.1 THz, 1.2 THz, and 1.3 THz. These features corresponds well to the simulation results of the THz transmission spectra (Fig. 1(d)).

**Figure 3:**
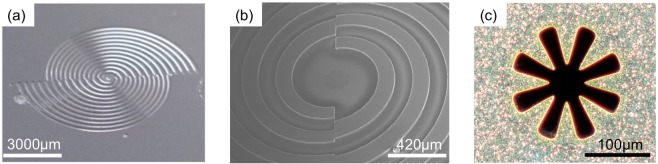
Images of the spiral plasmonic structure. (**a**) Photograph of the SBE structure and (**b**) microscope image of the double-corrugated devices. (**c**) Scanning electron microscope image of the eight-tip Siemens-star aperture at the centre of the SBE structure.

**Figure 4:**
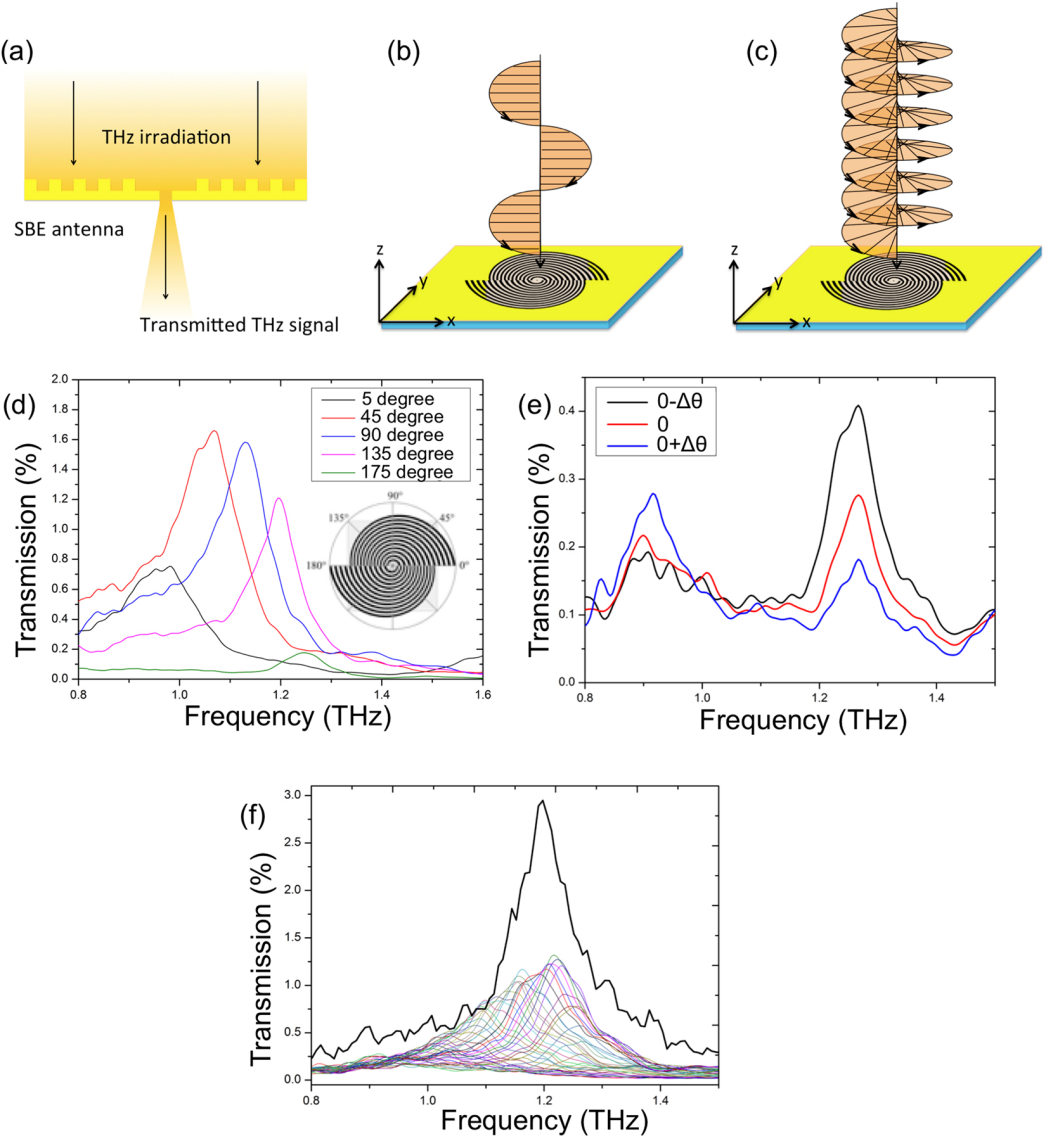
THz transmission measurement results of the SBE structure. (**a**) Illustration of the experimental set up used to measure the THz spectra. Schematics of (**b**) linearly and (**c**) circularly polarized THz irradiation onto the surface of the SBE structure. (**d**) Transmission spectra for the SBE grooves with different AoPs of the incident THz wave, showing five distinct highly tuneable transmission bands. (**e**) THz transmission spectra when the polarization direction of incident THz waves slightly changed around 0 degree. (**f**) The bold black line represents the transmission spectrum of the SBE for the circularly polarized THz irradiation. The colour lines correspond to the transmission spectra of the SBE for linearly polarized THz irradiation, where the AoP increased from 0 degrees to 180 degrees with a step size of 5 degrees.

Figure 3(a–c) display images of the fabricated SBE structure. Clear grooves and sharp edges are visible in both the photograph and the scanning electron microscope picture. To measure the spectral response of the SBE grooves according to the AoP, a linearly polarized THz wave was focused onto the *xy*-plane, as illustrated in Fig. 4(a,b). Figure 4(d) shows the measured AoP dependence for the THz transmission spectra of the SBE grooves. The AoPs studied here were set as 5° (nearly *x*-polarization), 45°, 90° (*y*-polarization), 135°, and 175° (nearly *x*-polarization) and were controlled by rotating the SBE grooves on the *xy*-plane. When the AoP of the THz incidence was almost parallel to the interface of the two half-SBEs (nearly *x*-polarization), the observed THz spectrum exhibited two transmission peaks at 0.95 THz (5°) and 1.25 THz (175°), whose positions were consistent with our simulation results. The transmission peak at 0.95 THz dominated when the AoP was 5° larger than 0°; in contrast, when the AoP was 5° smaller than 180°, the transmission peak at 1.25 THz was predominant. We also show more detailed transmission spectra of the SBE near 0° polarization in Fig. 4(e). Since this angle corresponds to the interface of the two half SBEs, the transmission frequency switches between ~1.25 THz and ~0.95 THz as the AoP passes through 0°: The peak at ~1.25 THz decreases and the peak at ~0.95 THz arises as the AoP approaches 180° (0° − Δθ), passes through 0°, and starts over from 0° (0° + Δθ). Thus, the relative difference in the peak height between the two resonance frequencies is sensitive to the AoP. The tuneable shift in the transmission peak with the AoP further confirms the behaviour expected from our simulations: when the AoP changes from 0° to 180°, the transmission peak position shifts from 0.95 THz to 1.25 THz, demonstrating highly tuneable transmission bands. This offers the possibility of freely selecting the plasmon-resonance frequency and amplifying the transmission intensity simultaneously by simply varying the AoP.

In addition to the unique characteristic of arbitrary frequency selection, the SBE also exhibits the subwavelength concentration of the circularly polarized THz field and a resulting enhanced transmission. The bold black transmission spectrum in Fig. 4(f) was acquired by directing a circularly polarized THz wave onto the SBE structure, as illustrated in Fig. 4(c). Both the intensity enhancement and bandwidth of the circularly polarized transmission were larger than those of the linearly polarized transmission, as the circularly polarized transmission entirely covers each single linearly polarized transmission spectrum due to SSP excitation over all AoPs. This result means that a circularly polarized THz field is concentrated on a subwavelength scale. This feature is expected to be of great value for useful applications, such as spin excitation and detection in magnetic materials and sensing of chiralities in chemical materials.

**Figure 5:**
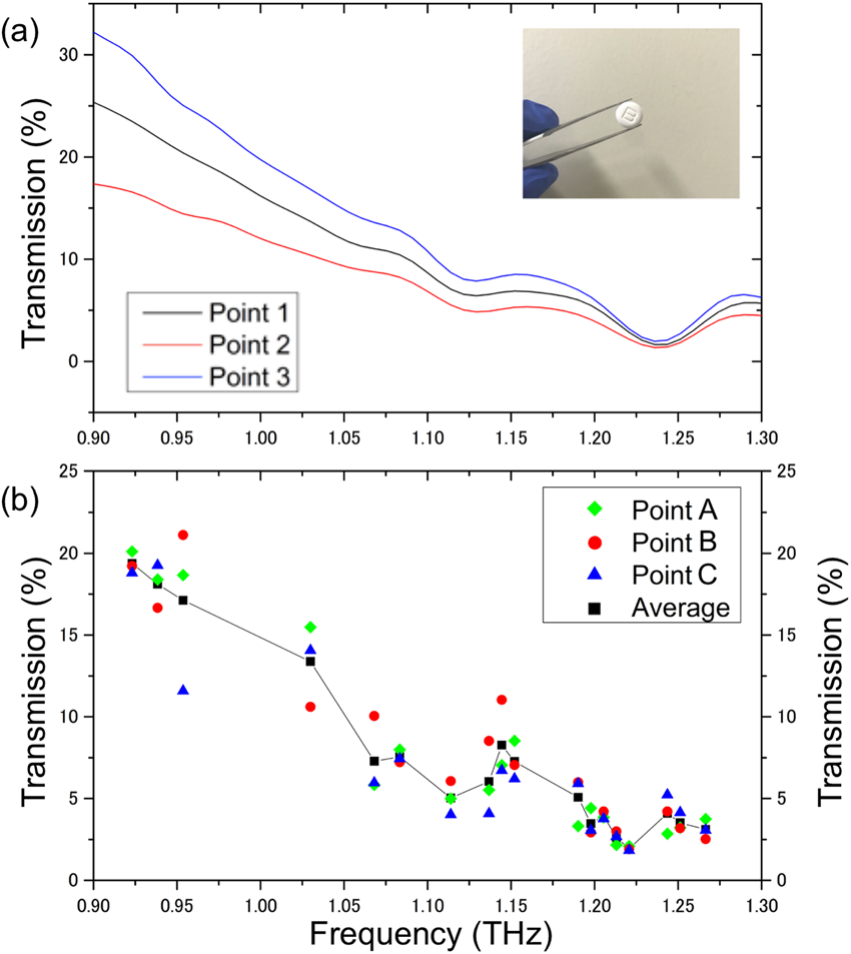
THz spectroscopic measurements with the SBE. (**a**) THz transmission spectra for a pharmaceutical tablet (bufferin) on the SBE. The data were taken at three different focus points, and the average of three data points was plotted together with a guideline. The AoP was varied by rotating the SBE. (**b**) THz transmission spectra for the same tablet as (**a**), measured by conventional mirror-based THz focusing (far-field measurement configuration). The THz spectra at three different points were measured. In (**a,b**), the difference in the THz spectra between the areas on the tablet is partly due to the inhomogeneous thickness of the tablet.

We then performed spectroscopic applications with the SBE. THz transmission spectra for a pharmaceutical tablet (bufferin) were measured through the interaction with the SBE to verify the availability of spectroscopic measurements based on the SBE. The tablet was placed near the SBE to ensure a subwavelength focus scale. The SBE was rotated every 10 degrees while the direction of the incident linearly polarized THz wave was kept unchanged. For comparison, THz transmission spectra at three different focus points on the tablet were tested using conventional far-field measurements with the THz-TDS, where the THz wave was simply focused onto the tablet via a parabolic mirror. Figure 5 shows the comparative results. The result for the use of the SBE clearly exhibited representative features (peaks and valleys) corresponding to those shown in the far-field measurement results. Moreover, since the focus size of the SBE measurement is much smaller than that of the far-field measurement with a focus diameter of 5 mm, the SBE enables spectroscopic measurements with higher concentration.

**Figure 6:**
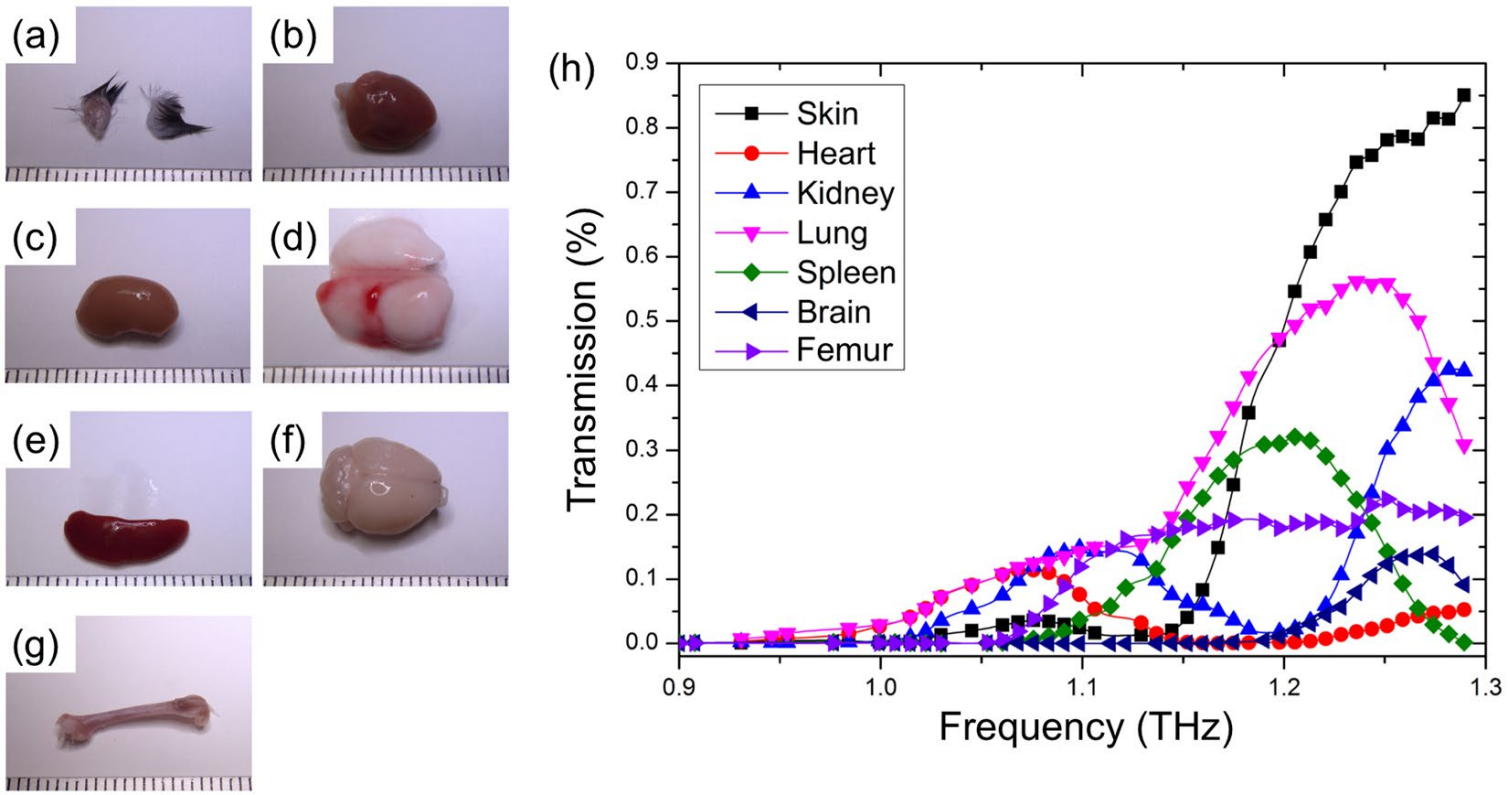
THz transmission spectra of bio-samples using the SBE. (**a–g**) The organs isolated from experimental mice for demonstrating bio-sensing: (**a**) skin, (**b**) heart, (**c**) kidney, (**d**) lung, (**e**) spleen, (**f**) brain, and (**g**) femur (Scale bar = 25 mm). (**h**) THz medical examination of sections of mice organ tissues for (**a**–**g**). The thickness of the cut sections was 500 μm. Transmission spectra were measured by rotating the SBE. The spectra revealed different transmission peaks characteristic of the organ tissues.

**Figure 7:**
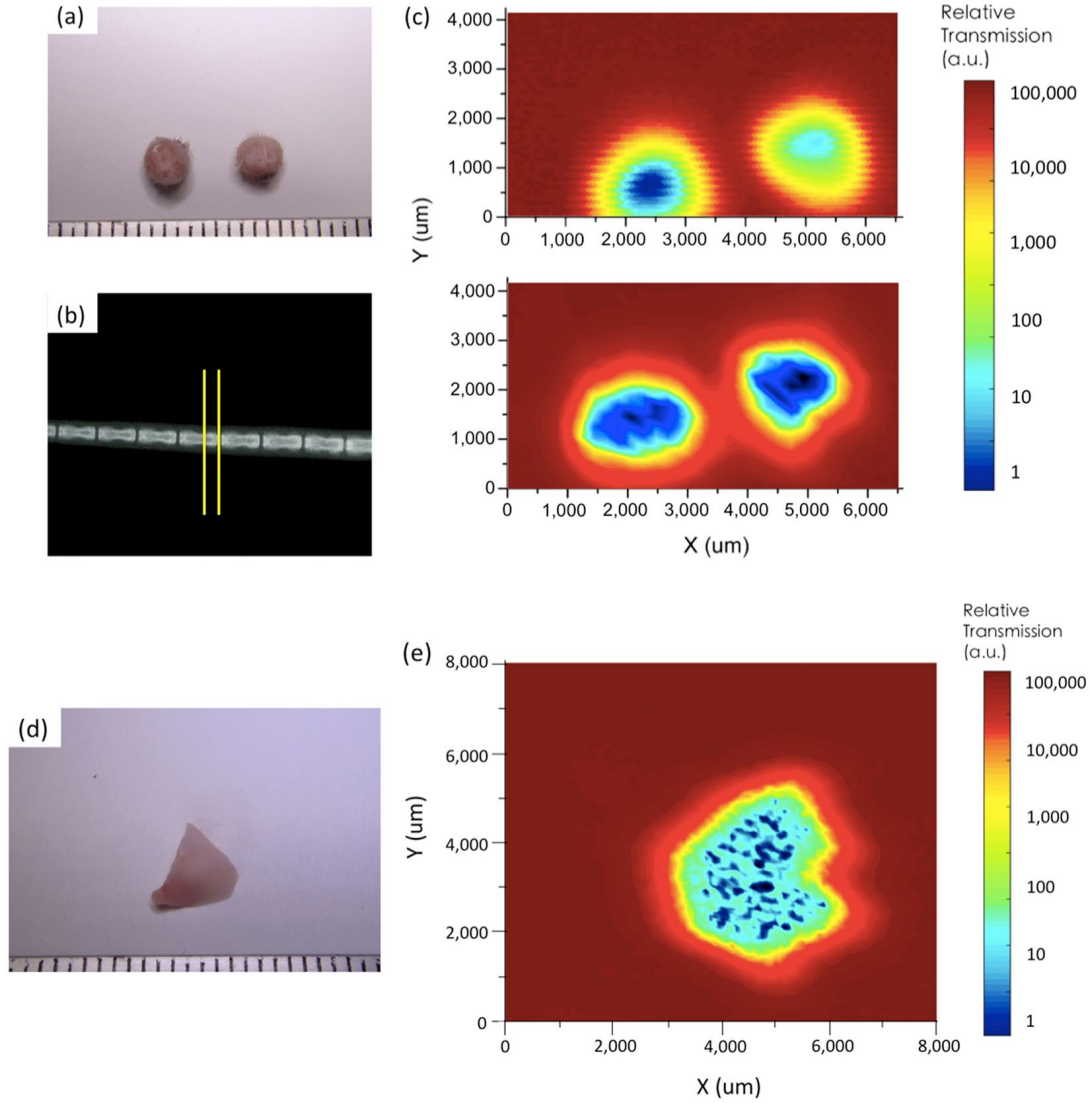
THz mapping of bio-samples through the SBE. (**a**) Mice tail-transverse sections with a thickness of 1 mm. (Scale bar = 25 mm) (**b**) X-ray picture of the mouse tail, with yellow lines indicating the cut point. (**c**) THz mapping of the mouse-tail transverse sections with a diameter of 2.5 mm using a conventional mirror-based focusing setup (upper image) and the SBE (lower image). In the SBE-coupled scanning result, hair (yellow and red), skin (light blue), and bone (dark blue) were clearly visualized. In the measured result without using the SBE, the image cannot be used to distinguish between different tissues and exhibits a much lower resolution. (**d**) Right middle lobe for THz bio-mapping. (Scale bar = 25 mm) (**e**) SBE-based THz imaging for the right middle lobe of (**d**). Three-dimensional biological tissue microstructures were visualized in it.

The frequency-tuneable and high concentration features of the SBE can also be useful for medical inspections. We studied THz spectra and images of bio-samples through the coupling with the SBE. Organs of an experimental mouse were cut (Fig. 6(a–g)) and put onto the SBE. Figure 6(h) exhibits THz transmission spectra for these cut sections. A frequency region between 0.9 and 1.3 THz is practical in medical sensing, as many characteristic peaks for bio-sample appear in this range. Most of the bio-samples did not show a high transmission between 0.9 and 1.0 THz, but showed transmission peaks at 1.06 THz, 1.1 THz, 1.2 THz, 1.25 THz, 1.26 THz and 1.3 THz. Transmission of the brain, heart, and bone were overall lower than that of other tissues, while the skin shows the highest transmission between 1.2 THz and 1.3 THz. Based on the large transmission difference between the skin and bone at 1.25 THz, we conducted THz mapping for the bio-samples by scanning them relative to the SBE surface. Figure 7(c) maps out the THz transmission image, where the sample studied contains two mouse-tail transverse sections with a diameter of 2.5 mm placed side-by-side. We compared the THz image taken by the conventional mirror-based THz focusing with that acquired using the SBE. The latter image (lower panel of Fig. 7(c)) clearly distinguished between hair, skin, and bone of the mouse tail, whereas the former mapping result (upper panel of Fig. 7(c)) exhibited only two blur shadows at the location of the bio-sample. We also carried out transmission mapping for a mouse-lung lobe using the SBE, as shown in Fig. 7(e). Biological three-dimensional microstructures were visualized in the middle of the lung lobe. We think that the observed dotted features might correspond to lung pipes. These results thus demonstrated that the SBE-based THz spectroscopy and imaging are of great value for medical examination.

In conclusion, we proposed and fabricated a continuously frequency-tuneable spiral plasmonic structure for multi-frequency THz analysis. Compared to other tuneable transmission devices^22–24^ that require either a change in the lattice constant or Fermi level of the materials, the SBE plasmonic grooves with a Siemens-star aperture provides a more convenient method based on simple rotation of the substrate of the SBE structure. Moreover, the spiral plasmonic structure not only works as a tuneable THz device but also enhances the electric field intensity at the subwavelength aperture. The SBE also can concentrate the circularly polarized THz field at the aperture. We further explored useful applications for the SBE in medical examination by measuring local transmission spectra of a pharmaceutical tablet and organ tissues through evanescent coupling with the SBE. The results confirmed plasmon-mediated sensitivity enhancement and frequency tuneability, enabled by the SBE. The THz mapping of mouse-tail transverse sections further verified that the SBE can be used to realize high-contrast, frequency-selective measurements with high THz field concentration. This structure can also be modified to fit most frequency ranges for SSP generation by changing the period and depth of the grooves^18^, and is, therefore, potentially useful for future wide-band, frequency-selective THz applications including nanomaterials characterization, chemical spectroscopy, and biological analysis. In particular, because THz-based medical diagnostic devices are highly promising as future medical care tools^25–31^, our SBE device is anticipated to be utilized as an important component for THz focusing and frequency tuning. The capability of circularly polarized THz field concentration and enhancement also provides an interesting possibility of investigating spin dynamics of magnetic materials, chiralities of optical isomers, etc.

## Methods

### SBE fabrication process

We chose to use a high-resistivity single-crystal silicon (Si) substrate due to its high transparency at THz frequencies. The spiral plasmonic grooves were microfabricated using deep reactive ion etching (DRIE) with a height of 30 μm on the ~1 mm thick Si wafer. Gold is one of the most widely used metals on the surface of BE structures since it provides a preferable surface for SSP propagation, leading to high transmission enhancement. However, our experiments showed that gold slowly diffused into the Si substrate, making the performance of the SBE structure less stable and thus weakening the SSP generation. Instead, we here used aluminium (Al) as the metal surface due to its excellent properties for generating SSPs and stable interface with Si, as well as its much lower production cost. The surface of the SBE structure was coated by an Al film with a thickness of 1.5 μm through sputtering. This procedure produced a double-corrugated structure with a plasmonic pattern surrounded by both the input and output facets of the apertures (Fig. 2(a,b)). Our measurements indicated that the 1.5-μm Al surface is thick enough to block the undesired penetration of incident THz waves with wavelengths between 200 μm and 300 μm, which guarantees effective SSP generation and propagation. Considering the Al thickness, instead of focused ion beam milling, we employed chemical etching with a solution of HF and H_2_O_2_ to define the eight-tip Siemens-star aperture. Figure S1 of supplementary materials schematically illustrates the fabrication process for the SBE grooves and aperture.

### Pre-measurements of the SBE structure

A broad-band THz-TDS system based on Cherenkov radiation, with a four off-axis parabolic mirror arrangement in a standard optical geometry, was used for spectral transmission measurements. The SBE device was positioned at the *xy*-plane, with the Si substrate normal to the *z*-axis. The THz-TDS measurements were performed in a nitrogen-rich environment. To verify SSP generation, we first plotted the time-domain signals for nitrogen and the SBE (see Fig. S2(a) of the supplementary materials). The SSP wave manifests itself as an additional waveform of the THz pulse response signal for the SBE, in contrast to the waveform of the THz signal for the nitrogen. Figure S2(b) of supplementary materials compares the transmission spectra between the SBE grooves and the single subwavelength aperture without such a plasmonic pattern. The spectrum observed for the SBE grooves clearly displays a large enhancement in the transmitted intensity, compared to that for the single subwavelength aperture.

### Bio-sample preparation

Four adult female C57BL/6 J mice (16–18 weeks old) weighing 15–20 g were used in this experiment. The animal experiment was conducted under approval (No. 0170218 A) by the Animal Care and Use Committee in compliance with the committee of the Tokyo Medical and Dental University, Japan.

#### Surgical organ isolation

The mice were anaesthetized with an intraperitoneal injection of sodium pentobarbital anaesthesia (100 mg/kg, ip) and transcardially perfused with 4% paraformaldehyde (PFA) in 0.2 M phosphate buffer. The thoracic cavity of the chest was exposed, and the skin, heart, kidney, lung, spleen, femur, tail and brain were extracted [Fig. 5(a–h), respectively] and then kept in 4% PFA liquid for no more than twenty-four hours.

All organs isolated from the experimental mice were sectioned along the vertical axis under a microscope, and the thickness of the section was 2 mm. The right middle lobe was peeled off for a separate experiment.

#### Sample packaging

The bio-samples were then removed and sealed between two pieces of transparent wrapping film with a thickness of 7 μm. This film can prevent the bio-samples from contamination and dehydration during measurements. The sealed bio-samples were fixed onto the SBE. In order to avoid undesired fluctuations, we also used a chilling facility and kept the temperature of the samples around 5°.

## Supplementary information

Supplementary information

Supplementary information

## References

[1] Clery D (2002). Brainstorming their way to an imaging revolution. Science.

[2] Nakajima S (2007). Terahertz imaging diagnostics of cancer tissues with a chemometrics technique. Appl. Phys. Lett..

[3] Ariyoshi S (2006). Terahertz imaging with a direct detector based on superconducting tunnel junctions. Appl. Phys. Lett..

[4] Kulesa C (2011). Terahertz spectroscopy for astronomy: from comets to cosmology”. IEEE T. on Terahertz Science and Technology.

[5] Kawano Y (2013). Terahertz waves: a tool for condensed matter, the life sciences and astronomy. Contemporary Physics.

[6] Vitiello MS (2012). Semiconductor nanowires for highly sensitive, room-temperature detection of terahertz quantum cascade laser emission. Appl. Phys. Let..

[7] Cai X (2014). Sensitive room-temperature terahertz detection via the photothermoelectric effect in graphene. Nat. Nanotechnol..

[8] Thio T (2001). Enhanced light transmission through a single subwavelength aperture. Opt. Lett..

[9] Nazari T (2014). Polarization dependent enhanced optical transmission through a sub-wavelength polygonal aperture surrounded by polygonal grooves. Opt. Exp..

[10] Nazari T (2015). Enhanced optical transmission through a star-shaped bull’s eye at dual resonant-bands in UV and the visible spectral range. Opt. Exp..

[11] Baragwanath AJ (2011). Terahertz near-field imaging using subwavelength plasmonic apertures and a quantum cascade laser source. Opt. Lett..

[12] Nazarov M, Coutaz JL (2011). Terahertz surface waves propagating on metals with sub-wavelength structure and grating reliefs. J. Infrared. Milli. Terahz. Waves.

[13] Gordon R, Brolo AG, Sinton D, Kavanagh KL (2010). Resonant optical transmission through hole-arrays in metal films: physics and applications. Laser & Photon. Rev..

[14] Maier, S. *Plasmonics: Fundamentals and Applications Ch 3*. (Springer, 2007).

[15] Zhang Z, Long Y, Ma P, Li H (2017). Tunable high-channel-count bandstop graphene plasmonic filters based on plasmon induced transparency. Nanotechnology.

[16] Zhang Z, Zhang L, Li H, Chen H (2014). Plasmon induced transparency in a surface plasmon polariton waveguide with a comb line slot and rectangle cavity. App. Phys. Lett..

[17] Wood JJ (2012). Spoof plasmon polaritons in slanted geometries. Phys. Rev. B.

[18] Deng X, Oda S, Kawano Y (2016). Frequency selective, high transmission spiral terahertz plasmonic antennas. Journal of Modeling and Simulation of Antennas and Propagation.

[19] Ding L, Xu W, Zhao C, Wang S, Liu H (2015). Coupling of plasmon and photon modes in a graphene-based multilayer structure. Opt. Lett..

[20] Ishihara K (2006). Terahertz-wave near-field imaging with subwavelength resolution using surface-wave-assisted bow-tie aperture. Appl. Phys. Lett..

[21] Bulgarevich DS, Watanabe M, Shiwa M (2012). Single sub-wavelength aperture with greatly enhanced transmission. New Journal of Physics.

[22] Liu P (2012). Tunable terahertz optical antennas based on graphene ring structures. Appl. Phys. Lett..

[23] Pelzman C, Cho SY (2016). Plasmonic metasurface for simultaneous detection of polarization and spectrum. Opt. Lett..

[24] Su Z, Chen X, Yin J, Zhao X (2016). Graphene-based terahertz metasurface with tunable spectrum splitting. Opt. Lett..

[25] Fitzgerald AJ (2003). Catalogue of human tissue optical properties at terahertz frequencies. Journal of Biological Physics.

[26] Siegel PH (2004). Terahertz technology in biology and medicine. IEEE Transactions on Microwave Theory and Techniques.

[27] Pickwell E, Wallace VP (2006). Biomedical applications of terahertz technology. J. Phys. D: Appl. Phys.

[28] Yu C, Fan S, Sun Y, Pickwell-Macpherson E (2012). The potential of terahertz imaging for cancer diagnosis: a review of investigations to date. Quant Imaging Med Surg..

[29] Redo-Sanchez A, Laman N, Schulkin B, Tongue T (2013). Review of terahertz technology readiness assessment and applications. J Infrared Milli Terahz Waves.

[30] Shiraga K (2014). Characterization of dielectric responses of human cancer cells in the terahertz region. J Infrared Milli Terahz Waves.

[31] Ouchi T (2014). Terahertz imaging system for medical applications and related high efficiency terahertz devices. J Infrared Milli Terahz Waves.

