# Supplementary material for: Continuously Frequency-Tuneable Plasmonic Structures for Terahertz Bio-sensing and Spectroscopy
Source: Sci Rep. 2019 Mar 5;9:3498. doi: 10.1038/s41598-019-39015-6 (PMC6401124; doi:10.1038/s41598-019-39015-6)
Supplement: Supplementary file 1 — Supplementary information [file 41598_2019_39015_MOESM1_ESM.pdf]

Supplementary information

Continuously Frequency-Tuneable Plasmonic Structures for Terahertz Bio-sensing and Spectroscopy

Xiangying Deng,[1] Leyang Li[2], Mitsuhiro Enomoto[2], and Yukio Kawano[1,*]

[1] *Laboratory for Future Interdisciplinary Research of Science and Technology, Department of Electrical and Electronic Engineering, Tokyo Institute of Technology 2-12-1, Ookayama, Meguro-ku, Tokyo 152-8552, Japan.*

[2] *Department of Orthopaedic Surgery, Tokyo Medical and Dental University, 1-5-45 Yushima, Bunkyo-ku, Tokyo 113-8519, Japan*

**Corresponding author: kawano@pe.titech.ac.jp*

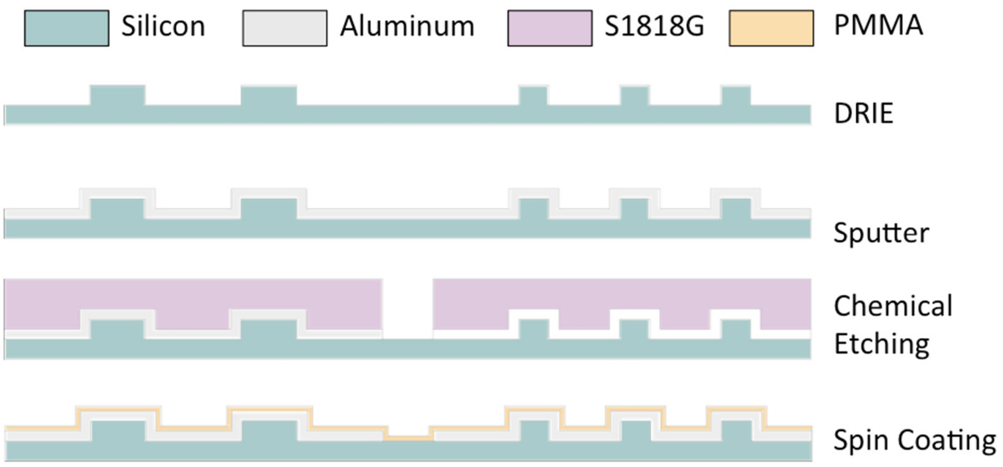

Figure S1 Fabrication process for the plasmonic structure with SBE grooves.

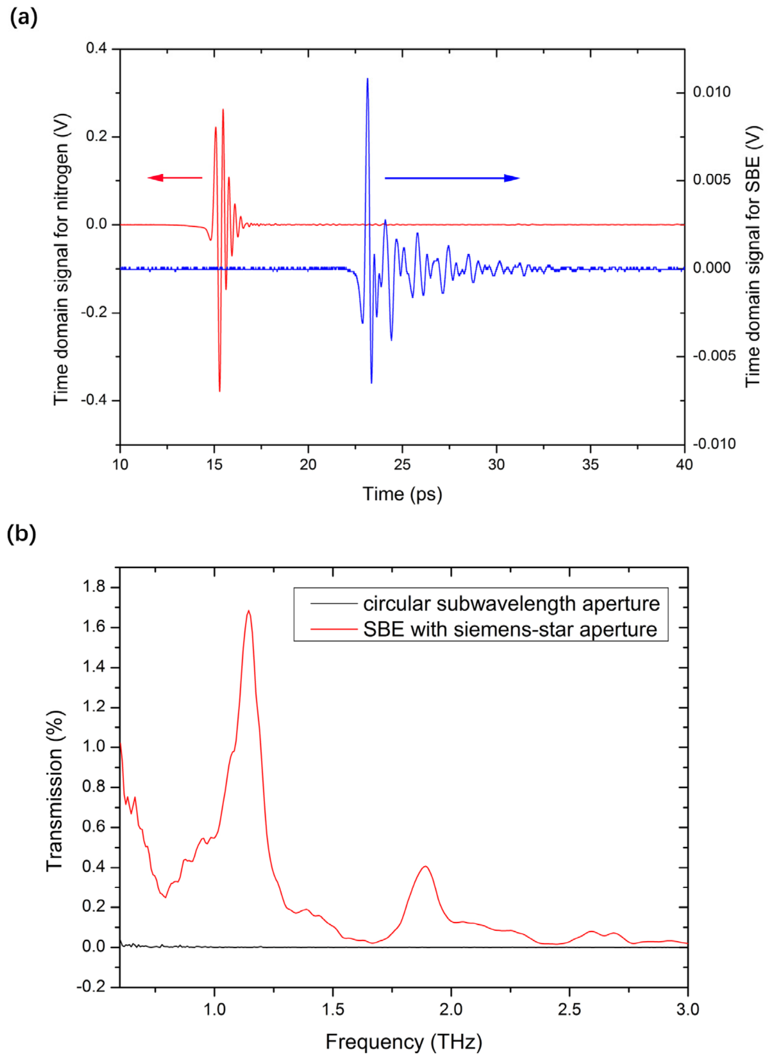

Figure S2 (a) Measured time-domain signals for the THz transmission through dry air (red) and the SBE structure (blue). (b) Comparison of the transmission spectra obtained for the SBE structure and Siemens-star aperture (red) and the reference substrate with only a circular subwavelength aperture without the plasmonic grooves (black). The AoP was set to 90°. The transmission spectra were obtained through Fourier transformation of the time-domain signals in (a).
